# Emergency Department Visits for Influenza A(H1N1)pdm09, Davidson County, Tennessee, USA

**DOI:** 10.3201/eid1805.111233

**Published:** 2012-05

**Authors:** Wesley H. Self, Carlos G. Grijalva, Yuwei Zhu, H. Keipp Talbot, Astride Jules, Kyle E. Widmer, Kathryn M. Edwards, John V. Williams, David K. Shay, Marie R. Griffin

**Affiliations:** Vanderbilt University, Nashville, Tennessee, USA (W.H. Self, C.G. Grijalva, Y. Zhu, H.K. Talbot, A. Jules, K.E. Widmer, K.M. Edwards, J.V. Williams, M.R. Griffin);; Veterans Affairs Tennessee Valley Health Care System, Nashville (W.H. Self, M.R. Griffin);; Centers for Disease Control and Prevention, Atlanta, Georgia, USA (D.K. Shay)

**Keywords:** influenza, pandemic, emergency medicine, cost of illness, viruses, Davidson County, Tennessee, A(H1N1)pdm09, pandemic (H1N1) 2009, pH1N1, H1N1, emergency department, United States

## Abstract

To determine the number of emergency department visits attributable to influenza A(H1N1)pdm09 in Davidson County, Tennessee, USA, we used active, population-based surveillance and laboratory-confirmed influenza data. We estimated ≈10 visits per 1,000 residents during the pandemic period. This estimate should help emergency departments prepare for future pandemics.

The 2009 pandemic influenza (H1N1) strain, hereafter referred to as influenza A(H1N1)pdm09, had the potential to substantially increase visits to emergency departments, many of which operate at or near capacity (*1**–**5*). Surges in emergency department patient volume cause treatment delays, low quality care, and increased risk for medical error (*6*). Understanding the number of visits associated with influenza A(H1N1)pdm09 should help emergency departments prepare for future influenza epidemics. We therefore estimated population-based emergency department visit rates attributable to influenza A(H1N1)pdm09 during the first year it circulated in Davidson County, Tennessee, USA. The Vanderbilt University Institutional Review Board approved this study.

## The Study

As part of the Influenza Vaccine Effectiveness network (Flu-VE) (*7*), we conducted active, prospective, population-based influenza surveillance among residents of Davidson County. We included those who had visited Vanderbilt University adult or pediatric emergency departments for acute respiratory infection (ARI) or fever/feverishness for <14 days during May 1, 2009–March 31, 2010. Nasal and throat swabs were tested for influenza with reverse transcription PCR (RT-PCR) by using primers and probes provided by the Centers for Disease Control and Prevention (Atlanta, GA, USA) (*8*). Specimens were classified as A(H1N1)pdm09 virus if results were positive on both pandemic subtyping assays (pandemic A and pandemic H1) or positive for influenza A, negative for seasonal subtypes H1 and H3, and positive on 1 pandemic subtyping assay.

We obtained the number of emergency department visits associated with ARI or fever (International Classification of Diseases, Ninth Revision, Clinical Modification, codes 381–382, 460–466, 480–487, 490–493, 786, and 780.6) from the Tennessee Hospital Discharge Data System (HDDS) (*9*), which is required to include a record of every hospital-based health care encounter. We combined data from Flu-VE RT-PCRs, influenza test results obtained clinically in the surveillance emergency departments, and HDDS discharge diagnoses to calculate age-specific visit rates attributable to influenza A(H1N1)pdm09. We used 2 epidemiologic methods: surveillance sampling and capture–recapture.

For surveillance sampling, we enrolled 826 (52%) of 1,589 eligible patients in the Flu-VE study who had visited surveillance emergency departments; 88 (11%) had positive RT-PCR results for A(H1N1)pdm09 virus (Figure). We divided the pandemic period into 3 intervals according to prevalence of A(H1N1)pdm09 among Flu-VE participants: prepeak (May–July 2009), peak (August–November 2009), and postpeak (December 2009–March 2010). Within each period, we assumed that the proportion of ARI- or fever-associated visits caused by A(H1N1)pdm09 virus among enrolled county residents was the same as that for such emergency department visits among all county residents. Estimated influenza A(H1N1)pdm09–associated emergency department visits were thus calculated by multiplying age- and time- specific counts of total county ARI- or fever-associated emergency department visits by these proportions (Table 1). We divided age-specific counts by age-specific county population estimates for July 2009 (*10*) and calculated rates per 1,000 residents (Table 2). We used the binomial Wilson method to calculate 95% CIs for the proportions of ARI- or fever-associated emergency department visits caused by A(H1N1)pdm09 virus.

**Figure:**
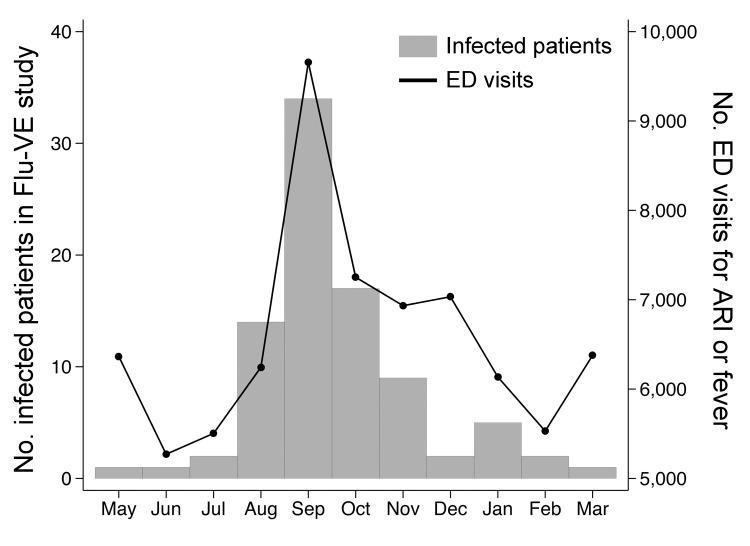
Number of patients enrolled in the Influenza Vaccine Effectiveness study at Vanderbilt University (Nashville, Tennessee, USA) who had laboratory-confirmed influenza A(H1N1)pdm09 virus infection (bars) and number of emergency department (ED) visits associated with a discharge diagnosis of acute respiratory illness (ARI) or fever (line) among all residents of Davidson County, Tennessee, May 1, 2009–March 31, 2010.

**Table 1:** Estimated total number of emergency department visits for influenza A(H1N1)pdm09, calculated by surveillance sampling method, Davidson County, Tennessee, USA, May 1, 2009–March 31, 2010*

Patient age, y	No. ARI or fever visits countywide†				% ARI or fever visits to surveillance emergency departments for influenza A(H1N1)pdm09‡			Estimated no. visits for influenza A(H1N1)pdm09 countywide
	Prepeak	Peak	Postpeak		Prepeak	Peak	Postpeak	
<5	3,135	6,461	5,219		0	19.0	0	1,249
5–17	1,599	5,269	2,324		15.0	24.0	0	1,528
18–49	7,322	11,523	10,486		2.4	23.0	6.3	3,455
>50	5,085	6,832	7,046		0	6.2	1.5	503

*ARI, acute respiratory infection. †Data from Tennessee Hospital Discharge Data System (*9*). ‡Data from Influenza Vaccine Effectiveness Study (Vanderbilt University, Nashville, TN, USA).

**Table 2:** Estimated emergency department visits for influenza A(H1N1)pdm09, Davidson County, Tennessee, USA, May 1, 2009–March 31, 2010*

Patient age, y	County population	No. visits/1,000 population (95% CI)	
		Surveillance sampling estimates	Capture–recapture estimates
<5	47,446	26.3 (15.2–42.7)	19.3 (11.0–38.3)
5–17	93,710	16.3 (8.90–27.9)	22.0 (12.0–46.5)
18–49	318,006	10.9 (7.48–17.2)	9.08 (5.67–22.6)
>50	176,548	2.85 (1.05–7.77)	3.60 (NC)
Total	635,710	10.6 (6.48–18.0)	10.2 (NC)

*NC, not calculated because of no matched cases in patients >50 years of age.

We developed a capture–recapture model (*11*) by linking 2 independent data sources for influenza testing from the same population: the Flu-VE RT-PCRs, performed in a research laboratory and not reported to patients or clinicians, and influenza tests performed as routine care in the surveillance emergency departments. Unlike the research laboratory tests, not all clinical tests included influenza A subtyping. However, all positive influenza A results were assumed to be A(H1N1)pdm09 virus because that strain circulated almost exclusively during the study period (*12*). To calculate the total number of influenza A(H1N1)pdm09–associated visits in surveillance emergency departments, we summed the following: the number of such emergency department visits detected by Flu-VE and clinical laboratory testing (a), the number detected by Flu-VE alone (b), the number detected by clinical testing alone (c), and the number missed by both systems (d). For each age group, we estimated the number of emergency department visits for influenza A(H1N1)pdm09 missed by both surveillance systems by using the nearly unbiased estimator equation, a modification of the Petersen estimator that performs well with rare outcomes: d = bc / (a + 1) (*11**,**13*).

RT-PCR identified 88 persons with influenza A(H1N1)pdm09; 541 patients had positive influenza A results by clinical tests: 506 BinaxNOW influenza rapid antigen tests (Alerei Inc., Waltham, MA, USA), 19 clinical RT-PCRs, and 16 viral cultures. Influenza A(H1N1)pdm09 virus was detected by clinical and research laboratory testing (“a” in the formula) for only 13 patients; age groups were <5 years (3 patients), 5–17 years (3), 18–49 years (7), and >50 (0). Using the nearly unbiased estimator equation, we calculated 572, 1,000, 528, and 90 surveillance emergency department visits for influenza A(H1N1)pdm09 for each age group, respectively. HDDS data indicated that 62.3%, 48.4%, 18.3%, and 14.2% of ARI- or fever-associated emergency department visits among county residents <5, 5–17, 18–49, and >50 years of age, respectively, occurred in surveillance emergency departments. We calculated the total number of influenza A(H1N1)pdm09–associated emergency department visits by county residents by dividing the number of influenza A(H1N1)pdm09–associated visits to surveillance emergency departments by the age-specific proportions above. To estimate rates, we divided estimated influenza A(H1N1)pdm09 visits by age-specific county populations for July 2009 (*10*) and multiplied by 1,000, yielding rates comparable to those obtained by the surveillance sampling method (Table 2). We calculated 95% CIs for capture–recapture estimates by using a bias-corrected bootstrap method (*14*). Because no persons >50 years of age were identified by both surveillance systems, 95% CIs for this group and the entire population could not be calculated.

## Conclusions

Using 2 epidemiologic techniques for calculating rates, we found that ≈1% of the Davidson County, Tennessee, population had visited an emergency department for influenza A(H1N1)pdm09 during the first year of virus circulation. The study has several limitations. The reported rates are dependent on the sensitivity and specificity of influenza tests. Delays in seeking care could have resulted in some influenza A(H1N1)pdm09 cases being undetectable, and if so, rates reported here would underestimate true rates of influenza A(H1N1)pdm09–attributable emergency department visits. Because active surveillance activities did not influence the possibility of influenza identification through routine emergency department care, the independence of these systems was assumed for capture–recapture calculations. However, this assumption could have been violated in some instances, for example if influenza viral load varied substantially among persons and higher viral loads increased the likelihood of detection by both systems. In this scenario, our method would underestimate the true number of emergency department visits (by increasing the number of matched cases). The proportions of ARI- and fever-associated emergency department visits for A(H1N1)pdm09 virus infection were extrapolated from surveillance emergency departments to the entire county population. If this proportion were higher (or lower) in the surveillance emergency departments than in other emergency departments, our rates would overestimate (or underestimate) true rates. Additionally, the small number of cases detected in adults >50 years of age precluded further age stratification among older adults. Because this study was conducted in an urban US county with high accessibility to emergency departments, we advise caution when extrapolating our estimates directly to other populations.

A modern influenza pandemic of mild severity can quickly cause large surges in emergency department visits. To minimize emergency department overcrowding and to maximize efficient use of resources, long-term preparation for these surges is vital. The high number of emergency department visits during the pandemic also illustrates the large effect a novel influenza stain can have on an unvaccinated, susceptible population and highlights the need for continued influenza vaccine development and use.
